# Intimate partner violence in the Canadian territorial north: perspectives from a literature review and a media watch

**DOI:** 10.3402/ijch.v72i0.21209

**Published:** 2013-08-05

**Authors:** Pertice Moffitt, Heather Fikowski, Marshirette Mauricio, Anne Mackenzie

**Affiliations:** 1 Health Research Programs, Aurora Research Institute, Yellowknife, Canada; 2 Aurora College, Yellowknife, Canada

**Keywords:** intimate partner violence, domestic violence, Canadian North, media watch, literature review

## Abstract

**Introduction:**

Family violence is a complex, multidimensional and pervasive presence in many Aboriginal communities. Although practitioners acknowledge that intimate partner violence (IPV) is a grave concern in the North, as in other jurisdictions in Canada, there is a paucity of literature about IPV and the local response to that violence.

**Objective:**

The purpose of this study is to report on a synthesis of Northern Territorial literature and a 3-year media watch conducted in the Canadian territories.

**Design:**

This review is part of a multidisciplinary 5-year study occurring in the Northwest Territories (NT) and northern regions of the Prairie Provinces of Canada. The methods included a review of the literature through CINAHL, PubMed, Academic Search Complete, Social Sciences Index and JSTOR (1990–2012) combined with a media watch from 2009 to 2012. A thematic content analysis was completed.

**Results:**

Themes included: colonization; alcohol and substance use; effects of residential schooling; housing inadequacies; help-seeking behaviors; and gaps within the justice system. Identified themes from the media watch were: murders from IPV; reported assaults and criminal charges; emergency protection orders; and awareness campaigns and prevention measures.

**Conclusion:**

When synthesized, the results of the literature review and media surveillance depict a starting context and description of IPV in the Canadian territories. There are many questions left unanswered which build support for the necessity of the current research, outline the public outcry for action in local media and identify the current published knowledge about IPV.

Violence is intolerable and destructive to individuals, families, communities and to all societies. In the Canadian territories, as in other parts of the circumpolar world, intimate partner violence (IPV) is, unfortunately, well known to the people who live there. The effects of IPV are discussed publicly by local activists in the hope of obliterating its occurrence and yet whispered behind closed doors in some remote locations where relationships are intimately bound. The variables that increase the incidence of IPV are intermingled with the support, or lack thereof, as well as the uniqueness of Aboriginal and remote communities. Furthermore, these confounding variables as well as the micro and macro effects of IPV are traumatic and intersect private and public domains. There is a paucity of published academic literature about IPV in the Canadian north, but stories of charged perpetrators, victims and fatalities appear in abundance in the local newspapers. For that reason, the methods in this study combine a focused literature review with a media watch from 2009 to 2012.

Similarities do exist between circumpolar countries especially in rural and remote areas where there are rugged terrains in isolated locations, arctic temperatures, isolation, small populations and limited resources. For that reason, the experience in the northern Territories of Canada may resonate with the IPV experience in other countries. The impact of the western world colliding with traditional values and lifeways along with colonization has disenfranchised people. These experiences may be felt across the circumpolar world. Furthermore, there is a need to explicate rural and remote context and experiences since the more prevalent urban interventions do not fit in the northern regions, which are distinctly different.

This study begins with a background to both the definition of IPV and a context to the unique northern region within which services are provided to women and their children experiencing IPV. A description of methods utilized within the review is provided. The results of the systematic literature review and media watch are identified as well as considerations for practice, policy, research and education in the Canadian North.

## Background

IPV is defined as coercive, harmful and abusive behavior, such as physical, sexual, emotional and/or psychological abuse, by a current or former partner within an intimate relationship (1). This includes the existence of and/or threat of future violence. Other terms commonly used include domestic violence, spousal abuse and women abuse.

Understanding IPV as a health and social issue requires exploring the problem in context (2). The regions within the vast territorial Canadian North, including Yukon (YT), Northwest Territories (NT) and Nunavut (NU), have their own unique geographic, economic, political and cultural features that are important to understand when considering the lived experience of women and their families who encounter IPV (3,4).

The territories have a significantly lower population in comparison to the provinces in Canada, which contradicts the incredibly vast geographic area that these 3 territories encompass. In 2011, the population of YT was 33,897, NT was 41,462, and in NU it was 31,906 (5). To further compound the low population base, the distribution or residents is scattered over a large geographical area into many small and remote communities. A number of the communities cannot be accessed by road and are solely supported by fly-in services or, where possible, seasonal ice roads. This geographical isolation with inherent limited services means that women experience IPV differently. It also poses many challenges in the service response to IPV, such as access and availability of formal resources.

For example, in the NT there are 33 communities but at this time, only 5 have emergency housing shelters for women and families fleeing abusive situations. Also, these few shelters are not always able to provide services due to funding and/or staffing shortages. In addition to the paucity of shelter services, many women are required to leave their home communities to access assistance. This isolates them, not only from family support, but also from their culture. In addition to a severe lack of shelter services, 11 communities do not have Royal Canadian Mounted Police (RCMP) detachments. Subsequently, a violent incident in one of these communities may have women waiting hours for authorities to arrive.

The low community populations within the NT, often only a few 100 people, have additional complexities to women experiencing IPV beyond a lack of formal services, or their less frequent use of them. The very short web of relationships within a community makes it difficult and uniquely complex in terms of accessing formal supports and/or services. For example, the abusive partner may be a relative of an important community leader or service provider, such as the local band council, employed at the Health Centre or local airport. These intersecting relationships can further complicate a woman's experience, willingness or ability to access services. It asks the question if indeed it may add to her fear, silence and success in fleeing a violent relationship.

Aboriginal people encompass a huge proportion of the territories, with First Nations, Métis or Inuit people living in 85% of NU, 51% of NT and 23% of YT. We acknowledge that IPV is a form of violence against women, Existing literature on IPV specifically denotes family violence, spousal or domestic violence or IPV as a serious issue faced by Aboriginal women and more generally, within Aboriginal communities (6–10). “Aboriginal people themselves say that the problem of family violence is so serious, that it has gone beyond hurting families” [to harming the entire community] (11). Campbell (7) suggests that the estimated high rates of violence in Aboriginal families are not reflecting an accurate depiction of violence occurring; we should assume the reality of violence to be considerably higher. This stems from acknowledging the slim chance that violent IPV incidents get reported to the authorities. One Canadian study looked at gender differences and socio-economic characteristics to IPV experiences (12). These researchers found a greater likelihood and increased severity of IPV experiences in women, and particularly those who are also Aboriginal, younger, with a lower annual household income, lower education, economically dependent and raising young children.

## Methods

The electronic databases used for this literature review included CINAHL with Full Text, PubMed, Academic Search Complete, Social Sciences Index and JSTOR using 2 different search engines Proquest and EBSCOhost (1990–2012). We also included a review of government documents, local reports and 2 dissertations relevant to the project. Keywords used included: domestic violence, family violence, IPV, spousal assault, battered women, violence against women and conjugal violence in the YT, NT, NU and Northern Canada. A secondary search through references within articles that met our selection criteria was also completed. Articles were selected if they met 2 particular criteria. First of all, articles were included that reported on research (both qualitative and quantitative) results about IPV. Second, articles were included that provided results that occurred in the 3 territories and Northern provinces.

Since there is very little published research from the Canadian Territories, we saw merit in expanding the accumulated data to include local sources of information and a review of media from 2009 to 2012. This included news and online reports from the Canadian Broadcasting Corporation (CBC) North and the 2 territorial newspapers. Keywords were used to search on-line news as well as a real-time surveillance of news reports. Newspaper articles were printed, collated and categorized by a central topic. Although non-academic, these documents add a context to what is publicly known or perceived about incidents in the Canadian North. From these, themes were assigned based on analysis of media collected over the 3-year period.

### Ethical approval and research license

This project has received ethical approval from the Research Ethics Board at the University of Regina and a research license from the Aurora Research Institute.

## Systematic review of literature

In Canada, most IPV research has been concentrated within more southern and urban locations; little work has highlighted rural areas and/or the Canadian North. This changed in 2004 when Statistics Canada's General Social Survey (GSS) piloted a survey on victimization and collected data from residents in the NT, NU, and YT (13). Though somewhat under-studied, several themes emerged within this literature review including colonization, alcohol and substance use, effects of residential schooling, housing inadequacies, help-seeking behaviors and gaps within the justice system.

### Colonization

Among Aboriginal people in Canada, family violence is a social issue that evolved as a consequence of social injustices and cultural oppression experienced with colonization (14). The high incidence rates of IPV with Aboriginal women (15,16) and in Aboriginal communities have been closely linked to historical and prevailing colonization (17). The 1999 GSS reported 21% of Aboriginal people in Canada, as compared to 6% of non-Aboriginal people, experiencing some form of physical or sexual violence by an intimate partner within the last 5 years (18). Furthermore, the 2009 GSS reported IPV experiences were more common among Aboriginal people in Canadian territories; 1 in 10 residents reporting spousal assault had contact with that former or current partner.

Colonization not only transformed the geographical location of Aboriginal people but also shifted gender regime away from males, most obviously in the mid-1970s (19). For example, IPV rates among the Inuit in NU were the highest in Canada and the rate seemed higher after they were resettled from the land into hamlets (15). Disproportionate rates across the 3 Canadian territories were also reported in the 2009 GSS. The NT and NU reported 12 and 14%, respectively as compared to a lower proportion found in YT of 6%.

The patriarchal nature of North American society was introduced with colonization. There was a shift from an egalitarian Aboriginal society, where men and women shared equal power in the subsistence economy, to the post-colonial society. Aboriginal men struggled to impose patriarchy amidst a socio-economic downturn as women became the main wage earners.

Post-resettlement, Inuit women were more likely to embrace schooling and became employed outside the home. Among the Dene people, women were more likely to attain higher education, training and become the wage earners as compared to their male counterparts. Additionally, men's more traditional wage-earning roles, trapping and hunting, were experiencing a decline. This led to a situation wherein men were threatening violence to women who were expressing an interest in advanced education. Within Aboriginal families, post-colonial changes led to a power shift between genders as well as within gender roles, and contributed to the low self-esteem among Aboriginal males. In addition to esteem, increased rates of alcohol and substance use, depression and suicide were seen in men. Similar rates were also seen in Aboriginal women and attributed to both the cultural changes since colonization as well as socio-economic factors such as family violence (19). Durst (20) attributed the unsettled change in gender relationships and IPV to factors such as the decrease in traditional activities due to industrialization, increased exposure to media, improved transportation, changes in female roles, and financial wealth. To sum up some of these direct colonial impacts, some Inuit families identified precipitators of IPV as economic stress and dependency, disagreement and jealousy, lack of communication between intimate partners, and alcohol and substance use.

### Alcohol and substance use

Korhonen (19) describes alcohol as a “fairly recent import” to residents of Northern Canada and suggests Arctic people have not developed the rules and rituals for drinking, unlike people from an “alcohol-adapted culture” (p. 37). Alcohol abuse was also suggested as the “single most important health problem in the circumpolar world” relating it not only to health effects but also to family violence (19). As presented in disparate ways within the literature, alcohol use played a role in the perpetration of IPV and violence against women as a root cause, an aggravating factor, or both.

Many women who experience IPV have histories of substance abuse; alcohol and drug abuse had been linked as both a predisposing and aggravating factor to IPV in Aboriginal communities (21). The increasing availability of alcohol and drug substances paralleled the peaking rates of domestic violence in the Canadian Arctic (3). In 2009, about 65% of IPV victims in the territories reported that their current or former spouse had been drinking during the violent incidents (13). Brownridge (6) acknowledged larger amounts of heavy alcohol consumption and IPV incidents with Aboriginal people, but suggested that this is reflective of a larger macro issue rather than a cause–effect relationship between them.

### Effects of residential schooling

The residential school experience of Canada's Aboriginal people has been identified as a contributing factor to IPV (21). The historical exposure to violence, trauma and abuse has created and sustained an intergenerational cycle of violence. As either a witness to violence or having direct experience with it as a child, Aboriginal adults are more susceptible to being a victim or an offender of IPV (22). Preventing Aboriginal children's exposure to family violence may be fundamental in ending violence among people in Canadian Aboriginal communities (23).

### Housing inadequacy

One critical issue that continuously challenged community response to IPV is housing. Housing inadequacy and overcrowding has been closely linked to IPV in Northern Canada (24). Additionally, residents of rural and remote northern communities had inadequate housing options as well as limits to readily available transportation and specialized services (21). For example, most women experiencing IPV never use shelters and/or find it difficult to afford social housing (24,25). In rural and remote northern communities, the options left for many abused women are either to continue living in their homes under the threat of violence or transfer to safer places in a community away from their families. As a consequence, family violence or IPV had been a major contributing factor to women's experience of homelessness (24).

### Help-seeking

In general, it is highly unlikely that families report violence that happens within the home or involve resources outside the home with what is usually considered a private matter (7,11). IPV had been underreported in Northern Canada for fear of retaliation by the abuser, financial and social dependence on the abuser, public ridicule, and concerns with the justice system process (11). Deeply rooted to the issue of underreporting is the normalization of violence in Aboriginal families (8).

Recent studies on help-seeking rates for IPV in Canada had shown that although Aboriginal women are more likely to be abused by their partners than non-Aboriginal women, the rates of help-seeking are similar (26). The likelihood of disclosing experience of IPV was found to be higher in the 1999 Violence Against Women Survey than that in 1993 (27). This changing pattern of help-seeking in women experiencing IPV coincides with increasing efforts to improve the criminal justice activity related to IPV.

### Gaps in the justice system

In the NT and YK, a civil legislation targeting family violence including IPV was initiated (28). The interventions included removing the offender from the home, permitting the victim to remain in the household with custody of the children, and implementing protective orders to prevent contact between victims and offenders (28). This is a fragmented approach. A deliberation on how women are positioned in society amidst colonialism and within an isolated northern environment with limited resources to endorse these interventions may constitute a more holistic policy direction. Paterson (28) concluded that policy frameworks not only failed to target the structural causes of violence but also scrutinized the behavior of women who experience IPV. In Canada's northern communities, social and economic conditions directly impact the lives of the residents and indirectly impact justice administration (29). An example of the indirect impact on administering justice is the reality that decision-makers in remote and isolated communities in northern Canada are from different cultural, social and economic background than the residents. There are significant cultural differences between the people in northern communities and those who are administering criminal justice system such as lawyers, probation officers, police, court staff and judges. Moorcraft (30) identified the loss of public trust by women in the YT with the police. Barrett et al. (31) suggested that the historical context of oppression and trauma faced by Aboriginal people negatively affected their interaction with the police and the criminal justice system.

## Synthesis of media reports

Although media reports may hold certain biases and/or personal opinions, they are a source of information that is useful to examine in terms of occurrences in the North and the response and reaction to these elicited events. Themes from the data include reported murders from IPV, reported assaults and criminal charges, emergency protection orders (EPOs) and awareness campaigns and prevention measures.

### Reported murders from IPV

Murdered women and children in the past 3 years have accounted for the deaths of 5 women and 2 children in the NT and NU in 5 violent incidents, respectively. The first homicide in this timeframe in 2009 was a Dene woman living in the remote community of Gameti, NT who was beaten to death by her husband. The perpetrator received a 7-year sentence for manslaughter. The NT Coroners report was released in 2012 with recommendations related to the death (32). Second, on June 13, 2011, an Inuk woman and her 2 young daughters (7 and 2 years) from Iqaluit, NU were found shot dead in their home; her spouse, the perpetrator, then took his own life at the grave of his sister in the local cemetery. Third, on December 19, 2011, an Inuvialuit woman was shot and killed by her partner in Tuktoyuktuk, NT. He then turned the gun on himself and ended his life. Fourth, and most recently, in June, 2012, a Metis woman in Fort Resolution was murdered in a domestic dispute. She went to the Health Centre in medical distress, was medevaced to Yellowknife and then Edmonton where she died. Her partner has now been charged with manslaughter after being found guilty a year previously of threatening her. Finally, in July 2012 in Hay River, NT a man murdered another man and woman. The Dene woman was said to have experienced prior abuse from the killer. The perpetrator had been charged earlier with assault of the victim and had served 30 days in jail. A common theme within these homicide accounts is that there was a history of abuse from the same perpetrator and that a prior criminal record was held.

### Reported assaults and criminal charges by partners

Although this is not a comprehensive review of the media, there were 15 assaults and criminal charges directly noted to be IPV reported in the Northern newspapers during this period of time. The contexts are varied but do provide descriptions of the nature of IPV in the territories. A few examples of particulars extrapolated from the clippings are included here that suggest that children are sometimes present; substance (drugs and alcohol) is a factor; holiday celebrations may be a factor; the perpetrators were often repeat offenders; weapons can include anything in the near vicinity of the outburst—telephones, fists, feet, knifes, rifles, fire; and in some cases family were called for assistance.

The captions are interesting and perhaps sensationalized to grab the reader's attention. One reporter wrote the following:

The woman told police that her spouse had forcibly held her down on a bed and “at one point forced her eye open” said Aitken. The woman crawled into the crib of the couple's 16-monthy-old son and was holding the child when her spouse tried to pull her out. When he couldn't, he slapped her across the face. Aitken told the court the man would not allow his spouse to leave or use the phone. She eventually was able to call her family in Inuvik and alert them to the situation. She then “hid in the bedroom until the police arrive” said Aitken. (33)

### EPOs

The media has also reported both satisfaction and dissatisfaction with EPOs. EPOs have been in place for close to a decade. EPOs were initiated in the NT to provide safety and protection from intimate partner perpetrators by inhibiting their access to their partner where there is evidence of abuse and violence. The RCMP enforces the EPO following judicial authorization. Some reports indicate that women are fearful of RCMP believing that RCMP do not always respond to the immediate needs of women or that when they do there is a prejudiced overtone to the response (30). One man in a public news account claimed that he had been wrongfully named as an abuser (34). A local politician took up the claim that EPOs are ineffective strategies in addressing violence (34). Lyda Fuller, community activist against family violence, asserts that EPOs are making a difference for many women and do decrease violence by restricting access (35).

## Discussion

The literature on IPV in the NT is scant, which is not unexpected since the location itself is mostly remote and under-resourced. However, it is this very remoteness and scarcity of resources that add to the complexity and the compounded effects that are experienced by families and whole communities who are intimately bound to each other. Although there is not an abundance of literature, researchers have focused on studying the aetiology, attributes, consequences of IPV and gaps in services. This broadens our understanding of some aspects of IPV. For example, knowledge is elucidated to identify influences of colonization, residential school and housing inadequacies on the creation of unhealthy environments. Whereby, violence culminates from suffering and pain too great to bear or where shame and blame become normalized in intergenerational dysfunction. Gaps in the service are also exposed. For example, the mental health needs of a colonized population and underserviced places are notably present but solutions are not readily available or identifiable. Researchers are skimming the surface of the issue as attempts are made to provide explanations of IPV in Northern Canada that lead to concrete efforts to care for residents and manage the effects of IPV. This is not enough. There is a need for a more comprehensive research agenda.

It is evident that social determinants of health, for example housing inadequacies, do not act in isolation, rather they are interwoven factors with poverty, unemployment, low literacy, etc. affecting territorial people and contributing to stressful living. In addition, the historical context of colonialism and male-dominated societies has created a volatile and oppressive milieu for women. Since the variables that appear to influence IPV are multifactoral, it is acknowledged that a comprehensive approach to both the investigation of IPV and the analysis of the data is required.

## Conclusion

This study has offered a targeted literature review of IPV in Canada's NT along with the results of a 3-year media watch. The themes from the published literature are contextually enhancing our understanding of both the IPV experience and the circumstances of those experiences. Colonization, alcohol and substance use, effects of residential schooling, housing inadequacies, help-seeking behaviors and gaps within the justice system are all explanatory to IPV. Media reports provide a perception of events surrounding homicides, assaults and charges to perpetrators as a result of IPV and also describe current interventions, such as EPOs and public awareness campaigns. The synthesis contributes to circumpolar knowledge of IPV.

It is important to note that there are existing resources provided in all 3 territories through government departments (victim services, shelters for women), and policing services. The Coalition against Family Violence, led by the NT Status of Women, has been instrumental in influencing policy direction with the government and in public awareness of zero tolerance to violence. Most recently, the Department of Justice in the NT has created a 9-month programme for male perpetrators of violence that is offered through the Healing Drum Society. Education sessions have been offered for police and front-line workers. Narrative therapy sessions are currently provided to assist counsellors. These activities are making a difference to local families.

This review illuminates the need for further investigation and action to eradicate violence in the North. Furthermore, the elevated number of IPV incidents indicates the need for establishing healthy relationships essential for healthy families, communities and societies, for productive and fully functioning growth and development, and for civility and peace within our homes and homelands. Finally, we can surmise that if violence were eradicated the environment at all levels (family, community, country) would be more conducive to happy and whole communities.
